# Designing and implementing smart glass technology for emergency medical services: a sociotechnical perspective

**DOI:** 10.1093/jamiaopen/ooac113

**Published:** 2022-12-30

**Authors:** Zhan Zhang, Noubra Ashika Ramiya Ramesh Babu, Kathleen Adelgais, Mustafa Ozkaynak

**Affiliations:** Department of Information Technology, School of Computer Science and Information Systems, Pace University, New York, New York, USA; Department of Information Technology, School of Computer Science and Information Systems, Pace University, New York, New York, USA; School of Medicine, University of Colorado, Aurora, Colorado, USA; College of Nursing, University of Colorado, Aurora, Colorado, USA

**Keywords:** smart glasses, workflow, sociotechnical, emergency medical services

## Abstract

**Objective:**

This study aims to investigate key considerations and critical factors that influence the implementation and adoption of smart glasses in fast-paced medical settings such as emergency medical services (EMS).

**Materials and Methods:**

We employed a sociotechnical theoretical framework and conducted a set of participatory design workshops with 15 EMS providers to elicit their opinions and concerns about using smart glasses in real practice.

**Results:**

Smart glasses were recognized as a useful tool to improve EMS workflow given their hands-free nature and capability of processing and capturing various patient data. Out of the 8 dimensions of the sociotechnical model, we found that hardware and software, human-computer interface, workflow, and external rules and regulations were cited as the major factors that could influence the adoption of this novel technology. EMS participants highlighted several key requirements for the successful implementation of smart glasses in the EMS context, such as durable devices, easy-to-use and minimal interface design, seamless integration with existing systems and workflow, and secure data management.

**Discussion:**

Applications of the sociotechnical model allowed us to identify a range of factors, including not only technical aspects, but also social, organizational, and human factors, that impact the implementation and uptake of smart glasses in EMS. Our work informs design implications for smart glass applications to fulfill EMS providers’ needs.

**Conclusion:**

The successful implementation of smart glasses in EMS and other dynamic healthcare settings needs careful consideration of sociotechnical issues and close collaboration between different stakeholders.

## INTRODUCTION

There has been growing interest in using health information technology (HIT) to improve patient care and increase the efficiency of healthcare professionals. Key examples include smartphone- and tablet-based mobile health applications which can be used anywhere to augment clinical work even when healthcare professionals are on the move.^1^ However, these mobile applications have their inherent limitations, such as their overreliance on manual input and control, which can be burdensome and in turn, hinder their effective use in dynamic, hands-busy healthcare settings.^2^ Given those challenges, wearable technologies such as smart glasses—a computing device worn as conventional glasses—have gained increased attention in healthcare because they can overcome the issue of manual input through their hands-free user interaction features (eg, voice recognition or gestural control).^3^ This emerging technology allows data presentation on the see-through optical display, recording of images or videos through a front-facing camera, and teleconsultation using a videoconferencing platform, among many other functionalities.^4^ A recent addition to smart glass features is the augmented reality (AR) technique which can capture and process a user’s physical environment and augment it with virtual elements.^5^

Over the past decade, smart glass technology has been used and tested out in a variety of healthcare settings and clinical scenarios.^4^ For example, researchers have explored using smart glasses to broadcast surgeries to remote consultants,^6^ record encounters with patients,^7–9^ monitor patient status in critical care,^10^ and support patient management and triage during mass casualty incidents.^11–13^ Despite these prior studies, limited research attempted to investigate the use and application of smart glasses in time- and safety-critical medical settings, such as emergency medical services (EMS) or prehospital care.^14^^,^^15^ As pointed out by prior work, the “hands-free” capability of smart glasses makes this technology of interest to EMS providers, who are usually physically and cognitively preoccupied with high-acuity patients and have limited capability to use handheld computing devices in real time.^16^^,^^17^ In our research, we aim to design and develop smart glass applications and hands-free interaction mechanisms to support EMS work practices and reduce their workload.

Implementing HIT in complex healthcare settings is challenging. Failures of many HIT implementations are largely attributed to the lack of consideration of user needs, workflow, and human factors entailed in the technology.^18^^,^^19^ When such issues occur, users such as clinicians have to bypass the new HIT intervention, and adopt informal, potentially unsafe practices and workarounds that may lead to disruption in the workflow and cause patient safety issues.^20^ As a novel technology, smart glasses face similar challenges in user adoption and integration with the work system.^14^ Therefore, to ensure successful implementation, it is of utmost importance to investigate, identify, and address critical social, organizational, and human factor considerations for using smart glasses in the fast-paced, dynamic EMS context. Addressing these issues at the early stage of system design can help prevent potential HIT failures, that is, the technology does not work as intended or designed, or is not used as expected.

In this study, we employed Sittig and Singh's^21^ sociotechnical framework for HIT implementation and adopted a user-centered design study approach by engaging EMS providers in a set of participatory design workshops to elicit their perceptions, user needs, and concerns with regard to using and adopting smart glasses in their work practice. The results of our study revealed critical factors and user concerns that need to be adequately addressed to ensure success and safe adoption and use of smart glasses in dynamic and fast-paced medical settings such as EMS. We conclude this article by discussing the implications of our study for designing the smart glass technology to support EMS work.

## METHODS

### Research goal and data collection

This study was part of a large research effort that aims to iteratively design and develop a smart glass application for EMS providers to facilitate real-time patient data collection, integration, and sharing in the field. In particular, we were interested in identifying system requirements of smart glasses in supporting EMS work and examining key factors that could facilitate or hinder the successful use and adoption of this novel technology. To that end, we employed a user-centered design approach and conducted 4 participatory design workshops with EMS providers over the course of 2 months (November-December 2021). Participatory design is an effective user-centered approach to creating a process that supports both researchers and domain experts/field practitioners in achieving a common understanding.^22^ Researchers in HIT have been increasingly using this methodology to generate innovative and high-quality results.^23^^,^^24^

A total of 15 participants were recruited from 3 EMS organizations (Table 1). Two organizations (A and B) are hospital-based EMS agencies in an urban area in the US Northeast region while organization C is a fire-based EMS agency located in a rural area in the US mountain region. Our participants included both emergency medical technicians (EMTs) and paramedics with varying working experiences.

**Table 1.** Participant characteristics

Workshop no.	Participant ID	EMS agency	Occupation	Total year of experience in EMS
Workshop 1	P1	A	Paramedic and EMS Director	40+
	P2	A	EMT	5
	P3	A	Paramedic	10
	P4	A	EMT	5
Workshop 2	P5	B	Paramedic	10+
	P6	B	Paramedic	20
	P7	B	Paramedic	27
	P8	B	Paramedic	6
Workshop 3	P9	C	Paramedic	18
	P10	C	Paramedic and EMS Educator	7
	P11	C	Paramedic	9
	P12	C	Paramedic	16
Workshop 4	P13	B	EMT	6
	P14	B	EMT	2
	P15	B	EMT	5

**Figure 1.**
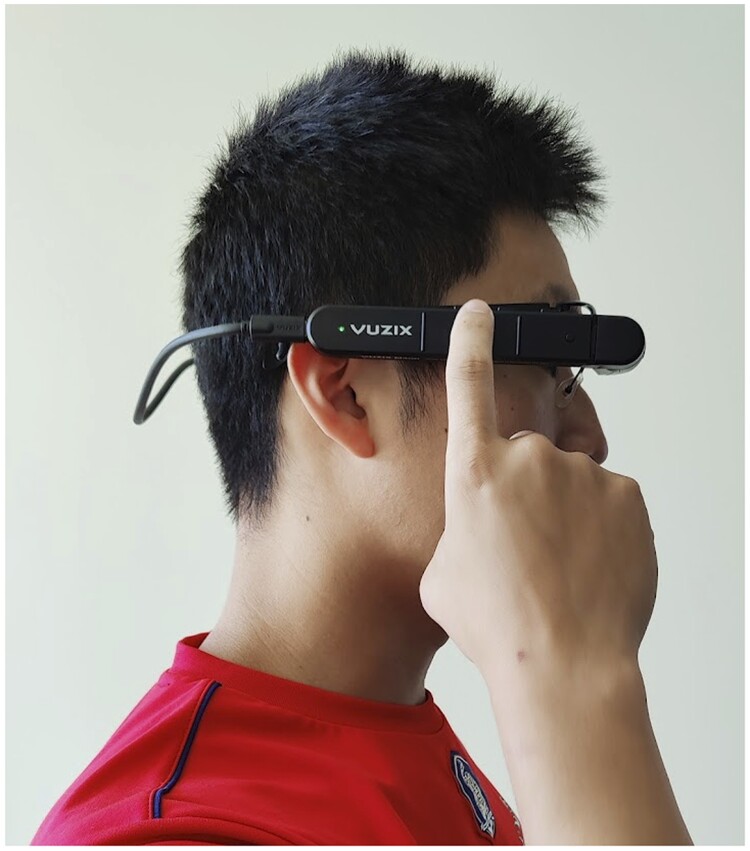
Vuzix M400 smart glasses used in the workshop.

Each design workshop consisted of 3 sections: storytelling, designing, and group discussions. In the storytelling section, study participants were asked to share their most recent patient care experience to “get into the mood” of thinking about the problem space. In the designing section, the participants were asked to discuss how smart glasses can help their work and create paper sketches to illustrate their design ideas. To help participants better understand the smart glass technology, we explained the major hardware and software components of the device and demonstrated different ways to interact with the device (eg, touchpad, voice commands, and hand gestures). We also demonstrated a system prototype that was developed in our prior work^15^ to illustrate how smart glasses can facilitate patient data collection and sharing in the field. For example, EMS providers can dictate patient information to smart glasses, and the dictation can be processed in real time to automatically populate the data fields in electronic health record (EHR). The prototype was built on the Vuzix M400 smart glass platform, which has a transparent optical display for presenting virtual content (Figure 1). In group discussion, we asked the participants to discuss key social, technical, and organizational considerations for using smart glasses in prehospital care. This discussion was guided by Sittig and Singh's sociotechnical framework,^21^ which has 8 interdependent dimensions representing the key aspects of HIT that must be considered to ensure their successful use and implementation (Table 2). Sittig and Singh’s framework has been widely used to analyze HIT-related issues and safety hazards.^25–27^

**Table 2.** Eight dimensions of Sittig and Singh’s sociotechnical framework^21^

Dimension	Description
Hardware and Software Computing Infrastructure	Physical devices, software, networking and storage devices, and communication infrastructure for supporting clinical work.
Clinical Content	Textual or numeric data and visual media data (eg, images and videos) that can be captured, entered, read, modified, stored, deleted, or used in the system.
Human-Computer Interface	The way users can see, touch, or hear as they interact with a system. Usability issues are also critical elements of this dimension.
People	Individuals involved in the design, development, implementation, and use of HIT, including users, system designers, and administrators.
Workflow and Communication	The processes or steps required in coordinating patient care tasks and communicating patient information.
Internal Organizational Policies, Procedures and Culture	Internal forces in an organization that influence the design, implementation, use, and adoption of a HIT intervention.
External Rules, Regulations and Pressures	Forces outside an organization that regulate, facilitate, or impede HIT implementation and use.
System Measurement and Monitoring	The process of measuring and evaluating system effectiveness, its use by users, and associated intended and unintended consequences.

This study was approved by Pace University Institutional Review Board. Consent was obtained from participants before each study session. The workshops were conducted remotely via Zoom and lasted 90–180 minutes. All activities were audio and video recorded.

### Data analysis

We transcribed the discussions and loaded the transcripts into Nvivo (QSR International, Version 12). Two researchers (first and second authors) first independently immersed themselves in the content of the data to get an overview. They then used an open coding technique^28^ to identify salient patterns and then classified them into the 8 dimensions of Sittig and Singh’s sociotechnical framework.^21^ Since the 8 model dimensions are interdependent, we classified our findings within the most representative dimension. In a subsequent step, 2 coders met regularly to compare results and discuss discrepancies. The third researcher was consulted when disagreements between 2 coders occurred. This process was repeated until all disagreements were resolved. Finally, we sorted the frequency of an opinion mentioned by our participants to determine their significance and relevance to EMS providers. This step allowed us to identify the most prominent sociotechnical considerations for smart glass implementation.

## RESULTS

In this section, we describe the critical factors and user concerns that emerged through our analysis organized by the 8 dimensions of Sittig and Singh’s sociotechnical framework.^21^ Exemplary quotations are presented in Table 3.

**Table 3.** Exemplary quotations representing our key findings

Dimension	Subcategory	Subcategory definition	Exemplary quote
Hardware-Software Computing Infrastructure	Device durability	Smart glass device needs to be durable and weather-proof to be used in dynamic and unpredictable environments.	The device needs to be weather-proof because we are outside in all conditions, rain, snow, sleet, etc. It must be destruction-proof as well. [P6]
	Battery duration	The battery needs to last long enough. With backup batteries, the device should run through a whole work shift.	These ambulances are running like, you know, nonstop for 24 hours. So, the charging might be an issue depending on the battery life. I don't know how easy it would be to swap a battery. [P8]
	Easiness of disinfecting	Easy to clean the device after each patient transport.	We get blood on it. Is it easy to clean? Like is there something protecting the button so it [blood] doesn't go inside the device? [P6]
	Network connectivity	Need a high-speed network to ensure reliable internet connections.	Another problem is connectivity. Wi-Fi doesn’t work, and sometimes network services don’t work great in the field or even in patients' homes. [P7]
	Interoperability	Interoperable with other computing and medical devices to ensure smooth data exchange among them.	Maybe integrate it with our monitor so we can directly pull the EKG off the monitor. [P6]
Clinical Content	Medication information	Scan medication barcode to automatically capture medication information (eg, name, timestamps, dosage, etc.).	It would be helpful to have the smart glass scan the barcode of the medication administered. But it should have a way for us to indicate the dosages. [P6]
	Contextual/Visual information	Take pictures or record videos of patient injury details for further use.	Sometimes we take photos using our phone and show that to ED physicians. But if we can do this and share the photos in real time, that would be even more beneficial. [P6]
	Dictate detailed patient information to smart glasses	Combine smart glasses and voice recognition techniques to automate data collection and documentation in the field.	If I can verbalize certain things, whether it's the vital signs or what you're doing, or what drug you're giving at a certain time, or what intervention you're doing, and if they could be processed through this smart glass and get documented as such, while your hands are free, that will save a lot of time. [P1]
Human-Computer Interface	Prefer hands-free interaction mechanisms	EMS providers prefer hands-free interaction mechanisms (eg, voice control and hand gestures) over built-in touchpad and buttons.	Voice control would be very good. I don't want to touch anything with my hands. A lot of times my hands are bloody, or I got vomit or urine, or I'll get all kinds of stuff on my gloves. [P11]
	Need minimal interface design	The interface of display needs to be designed intuitive and easy to use.	I guess the buttons and things on the screen all need to be configured to the least possible amount so that it [screen] does not restrict my vision. I would be bothered by having something in front of my eyes when I'm trying to look at a patient. [P11]
	Ergonomics issues	This type of issues represents concerns regarding whether smart glasses can fit with wearers’ PPE and eyeglasses, sit steady in front of wearers’ eyes, and not affect users’ vision.	Glasses might be falling off. We'll need to make sure that the frame has enough tension to hold on, especially when you're sweaty. [P9]
People	Impact on patients	Smart glasses can be intimidating to patients, especially pediatric patients.	It's kind of an intimidating thing to come in and film someone off the bat. Um, it can cause people to shut down or not open up to you. [P13]
	Training is needed	Tailored formal and refreshing training to EMS users is necessary.	I noticed a lot of bias to new technology; I think people are afraid of new technology. They must be trained to use it. [P8]
	Account for individuals’ differences in technology proficiency	The system design should take into consideration the various level of EMS providers’ technology proficiency and its impact on the use and adoption of smart glasses.	The EMS folks have different level of comfort with technology. Even in 2050, I’m sure there will be old timers that are only comfortable with using tablets, and there’ll be new kids who are comfortable using virtual crystals. [P15]
	Other stakeholders to involve	In addition to EMS providers, there are other types of stakeholders need to be involved in the system design process.	We are prehospital setting, the other end in the hospital should also be included. Like nurses who are triaging the patient or doctors who are dealing with the patient. [P13]
Workflow and Communication	Need to seamlessly align with EMS workflow	Smart glasses should be able to adapt to EMS workflow, rather than changing current workflow to adopt this technology.	We already have various digital systems in place. I think it's just important to envision this as another tool and it needs to fit into our work for constant use. [P15]
	Impact on teamwork and communication	Using smart glasses could potentially affect EMS teamwork and communication.	We are always like connected with our partners to work on things together. Like we are always moving the stretchers, grabbing bags or lifting patient. “You have that leg?” “Yes, I have the leg and you have the bag.” “Okay. 1, 2, 3, lift.” You know, we are always talking. So it’s gonna be really hard for me to break that kind of connection between me and my partner. You know, my partner may be waiting for me to answer something very important while I am busy operating the smart glass. [P14]
	Adapt to different types of EMS systems	The smart glass should be designed to adapt to different EMS systems and different regions in the United States	I think it is necessary to create a generic software that can apply to a lot of EMS systems. Each EMS system is unique and will likely request different tweaks to make it fit better with the systems that they already have in place. [P10]
Internal Organizational Policies, Procedures, and Culture	Obtain buy-in from key stakeholders	Need to obtain buy-in from key stakeholders to support the adoption of smart glasses both organizationally and financially.	It would depend on whether you get buy-in from our agency. I believe it is an expensive unit. Like, who is going to pay for that? [P5]
External Rules, Regulations, and Pressures	Compliant with HIPAA regulations	Smart glasses need to be complaint with HIPAA rules and regulations to ensure data security and patient privacy.	HIPAA would be hard to pass if you guys are unable to make that HIPAA compliant software, because as soon as we’re starting to transfer information back and forth between agencies, if that’s not a secure network, then our care is at risk. And we as an agency would be at risk for releasing, obviously private information and then causing a lot of issues. [P10]
	Medicolegal issues	Filming patient and bystanders could lead to potential medicolegal concerns	Sometimes we are in another facility, like one time, I had to defibrillator somebody in an outpatient surgery. If someone happened to walk through the background of my video that I was taking, would that be a security breach or something? Would they have to then sign something? Was I allowed to use it in their facility? [P5]
System Measurement and Monitoring	Critical to evaluating effectiveness and usefulness before and after system deployment	It is imperative to evaluate whether the smart glass technology is useful and efficient in supporting EMS work throughout the system implementation process.	When measuring effectiveness and usefulness, what I would see is a test group or a study group that would be supplying consistent feedback, documenting how many times it's been used, what conditions it was used in, or what situations they were using the software on. [P10]

### Hardware and software computing infrastructure

Our participants mentioned several major concerns about the hardware of smart glasses, including the device's durability (*n* = 10), easiness of disinfecting (*n* = 5), battery duration (*n* = 3), and network connectivity (*n* = 2). More specifically, the smart glass device’s durability is critical as EMS providers work outside of the hospital, exposing the computing device to dynamic and even extreme weather conditions; therefore, the device should be sturdy, antifog, water-repellent, and weather-proof to be used in an unpredictable environment. In addition, as EMS providers often constantly work outside in the field, the glass battery should last long enough. Our participants emphasized the need of having 1–2 backup batteries to make sure the device can run through a whole work shift. The easiness of disinfecting the device was also cited as a major concern, especially in the era of the coronavirus disease 2019 (COVID-19) pandemic—smart glasses can easily get contaminated while being used in the field and therefore, they need to be easily cleaned after each patient transport. Finally, since audio, video, and data transmission via smart glasses (eg, establishing video calls between EMS providers in the field and remote physicians) rely on a high-bandwidth network, whether it is possible to establish steady access to high-speed internet was considered a critical determinant for the uptake of smart glasses.

Concerns about the software component centered around the interoperability between smart glass applications and other medical and computing devices used by EMS providers in the field (*n* = 3). Interoperability is critical to ensure smooth data exchange between different systems. For example, our participants highlighted the importance of being able to transfer collected patient data (eg, pictures and video recordings) from smart glasses to their EHR device for permanent storage.

### Clinical content

Participants also discussed what types of clinical data can be collected by smart glasses for further use. For example, EMS providers need to capture and record some time-sensitive information, such as timestamps of treatments, as some medications may lose effect after a certain amount of time and EMS providers need to rely on accurate timestamps to determine whether the medication becomes effective or when to administer the next dose. Given this critical need, our participants (*n* = 8) saw an opportunity for smart glasses to facilitate the data collection of administered medication, that is, scanning medication barcode via the smart glass camera and associated software.

Another set of critical information that can be captured by smart glasses is contextual information (eg, injury details and severity) that is usually hard to describe. A few EMS providers (*n* = 6) envisioned the use of smart glasses in capturing visual patient information (eg, taking pictures of the patient’s trauma wound), which can be shared with the physicians in the receiving hospital to help them anticipate patient needs and allocate appropriate resources.

Lastly, many of our participants (*n* = 8) were highly interested in dictating to smart glasses to semiautomate EHR documentation because this feature, enabled by voice recognition techniques, could not only save significant time documenting detailed patient data in the field but also enable them to share the patient record with the receiving hospital before their arrival.

### Human-computer interface

Several design considerations regarding the interaction between users and smart glasses were discussed during the workshops. First, majority EMS providers (*n* = 12) would prefer using hands-free interaction mechanisms such as voice control or hand gestures to interact with the device; in contrast, the built-in touchpad and clickable buttons were indicated as the least preferred interaction mechanism, owing to concerns about cross-contamination. Second, as the screen of smart glasses is very small, our participants (*n* = 4) suggested that its interface design should be as minimal as possible to ensure the device is less obtrusive.

Several participants (*n* = 7) also voiced their concerns related to human factors and ergonomic issues while using the device. For instance, if the device does not hold properly on users' heads or is not compatible with users’ spectacles (eg, glass) or personal protective equipment (PPE), they might choose to not use the device at all. Another prominent concern was related to whether the device could block the user’s vision (*n* = 4). Even though smart glasses have a see-through, transparent display, users may still find the device cumbersome to wear when they are performing patient care.

### People

Regarding the people dimension, EMS providers (*n* = 5) highlighted the potential impact on their patients. For example, it is very likely that wearing smart glasses could intimidate patients, especially pediatric patients, who may have never seen this type of device or have no clue what this device is used for. Our participants, therefore, mentioned that they might choose to explain the purpose and benefit of using smart glasses if necessary.

Another essential aspect of this dimension discussed by a few providers (*n* = 3) is the necessity of providing tailored onboarding training to EMS providers to overcome user-related problems with this novel technology. Furthermore, the training needs to be offered regularly to “refresh” their knowledge about operating the device, especially after adding new or changing current system features.

### Workflow and communication

Our participants all agreed that smart glasses can potentially improve their current workflow. For example, as described previously, combining smart glasses and voice recognition techniques to facilitate documentation in the field could save EMS providers a tremendous amount of effort from manually entering detailed data entry into EHR so they can focus on patient care rather than the documentation task. In addition, smart glasses were believed to be able to enhance information sharing and care coordination between prehospital and hospital providers through “see-what-I-see” video communication.

Regarding the concerns of adopting smart glasses in their routine practice, of importance is the actual integration with current work practices and medical devices (*n* = 7). As EMS providers are already overwhelmed with many tasks and computing devices (eg, EHR), the smart glass application needs to fit into their workflow rather than drastically changing their current work practice. For example, EMS is a highly collaborative work environment with several providers contributing to patient care and data collection, who and how many providers would wear the smart glass device and how to process and integrate information collected by different providers need a thorough plan. Another concern raised by our participants (*n* = 3) is related to the impact of smart glass use on teamwork and communication. That is, using smart glasses demands wearers’ cognitive attentions, which could distract them from communicating with their partners. Lastly, there are varying types of EMS agencies (eg, fire-based, hospital-based, and volunteer-based) working in different areas (eg, rural area vs urban area). As some of our participants (*n* = 3) explained, this unique EMS characteristic requires that the smart glass system should be adaptable to different EMS systems and work contexts.

### Internal organizational policies, procedures, and culture

Only one issue was brought up within this dimension (*n* = 2)—for the EMS agencies to successfully adopt the smart glass, it is important to obtain “buy-in” from the leadership of the organization. That is because, as one of our participants explained, the EMS industry does not have strong financial support as other clinical settings; thus, obtaining “buy-in” from key stakeholders is of importance to secure financial support for purchasing new smart glass devices and associated software package, and covering the cost of training, maintenance, and replacement of damaged equipment.

### External rules, regulations, and pressures

As smart glasses would capture, transfer, and even store sensitive patient data, many of our participants (*n* = 10) emphasized the importance of making the device compliant with the Health Insurance Portability and Accountability Act (HIPAA) regulations. That is, all the recorded data should be encrypted. Also, the device should have advanced security mechanisms to ensure only authorized users can access the stored data, that is, in case the device is lost and picked up by a random person. In addition to data security concerns, some participants (*n* = 7) also discussed issues around using the smart glass camera for data collection or consultation as they could potentially lead to medicolegal concerns.

### System measurement and monitoring

It was a great consensus among our participants (*n* = 15) that continued efforts are needed to evaluate the effectiveness and usefulness of the system. It is more than necessary to iteratively test the smart glass application with simulations before field deployment. This effort could help the development team identify and address technical and usability issues and determine optimal ways to integrate the system into the EMS workflow. Our participants also shared several ideas regarding how to evaluate the system, that is, comparing handwritten notes versus automatically collected data by smart glasses to determine which method is more accurate and effective.

## DISCUSSION

In this work, we investigated the perspectives of EMS providers regarding the design and implementation of smart glass applications to support their work during prehospital care. Even though a few studies have examined the use of smart glasses by first responders in mass casualty incidents,^11–13^ they only evaluated the effectiveness of off-the-shelf smart glass devices (eg, Google Glass) on patient triage. However, there lacks a theory-guided investigation of how to design smart glass applications to support EMS work and what factors can facilitate or hinder their adoption by users. Aligning with prior work,^21^^,^^29^ we argue that it is critical to involve clinical users in the early phase of system design to ensure a successful HIT implementation. Applications of the sociotechnical model allowed us to identify a range of factors that influence smart glass adoption in EMS.

Our participants all agreed that the smart glass technology if working properly, has a huge potential to improve their workflow. For example, using the EHR system to document patient data in the field is a time-consuming and error-prone task.^30–32^ In particular, when EMS providers are physically preoccupied with patient care, they have limited capability to use handheld computing devices. Our participants agreed that combining smart glasses and advanced voice recognition techniques to automate documentation can significantly reduce their workload. In addition, in the United States, EMS providers currently rely on traditional telecommunication tools such as radio to communicate patient information with remote physicians for online medical control; however, this work practice is not effective as pointed out by previous studies.^33^^,^^34^ Prior work has demonstrated the usefulness of implementing telemedicine system into EMS workflow, such as enabling prompt and efficient patient treatment with optimized use of resources.^35^^,^^36^ Aligning with this line of research, EMS participants recognized the enormous benefit of using smart glasses to enhance communication and care coordination with the receiving care team.

To fully realize these benefits, our participants highlighted several key design considerations that need to be accounted for. Aspects related to hardware and software were seen as crucial for the uptake of smart glasses. Given that EMS providers often work outside of the hospital in diverse situations and environments, the device needs to be durable, weather-proof, and easy to clean. More importantly, steady internet access is a critical requirement for deploying smart glass systems in the field as they need a high-bandwidth cellular network to establish quality video and audio calls between the field and receiving hospital. Integrating 5G technology with smart glasses could be a viable solution. (https://www.vuzix.com/blogs/vuzix-blog/5g-to-expand-smart-glass-capabilities.) Another ongoing effort is building a dedicated broadband network for first responses (eg, FirstNet [https://firstnet.gov/about]), which could also improve the network connectivity for smart glasses in the future.

Another set of critical design considerations is related to the human-computer interface dimension. An intuitive, easy-to-navigate user interface with minimal design has been acknowledged as an important facilitator for implementation. This design requirement can help minimize the cognitive workload of using the system and reduce the likelihood of blocking users’ vision. Additionally, human factor issues such as whether smart glasses can be compatible with the user’s wearing and sit steadily in front of the user’s eyes even during excessive physical activities were also considered critical. This type of user concern was also brought up by early investigations of smart glasses in other healthcare settings (eg, surgical operations).^37^ Finally, our participants expressed interest in using the device in a hands-free manner because manually operating the device in the prehospital environment is impractical and could lead to cross-contaminations. Popular hands-free interaction methods such as voice commands and hand gestures^38^ could be employed. However, several key questions should be thoroughly investigated, such as whether voice commands can be accurately recognized in the noisy prehospital environment and whether gestural inputs have high social acceptance.^39^

Many providers in this study highlighted the importance of ensuring HIPAA-compliant data access, processing, and storage while operating with smart glasses. As our participants argued, this is one of the most critical requirements for adopting smart glasses in EMS work; any potential violation of HIPAA rules could make this technology another example of HIT failure. A related concern is that the device may get lost and picked up by a random person. A few participants mentioned this had happened to their EHR system and portable medical devices. To prevent unauthorized users from accessing the device, smart glasses should have advanced security mechanisms that only grant access to authorized users (eg, identifying the glass user through iris recognition^40^) Lastly, using smart glasses for data collection and sharing could raise medicolegal questions. For example, should EMS providers obtain patient’s consent before taking pictures? What if the patient resists using smart glass for teleconsultation? Who is responsible for inaccurate data collection caused by software malfunction?

Many failures of HIT implementation could be attributed to the misalignment between HIT design and actual clinical workflow.^41–44^ As such, our participants reiterated the importance of integrating smart glasses with their work practices and ensuring interoperability between smart glasses and existing computing and medical devices. One key example is ensuring smooth data exchange between smart glasses and vital sign monitors so that EMS providers can view and monitor patient’s vital signs via smart glasses even when they are moving around (eg, triaging multiple patients). In a similar manner, the device should be seamlessly integrated with the EHR system so providers can use smart glasses to chart patient records in a hands-free manner. Aligning with prior work on the impact of HIT implementation,^45^ our participants also pointed out the potential impact of smart glasses on teamwork; for instance, similar to using other computing devices (eg, EHR),^46^ wearing smart glasses could distract the users and affect their ability to tacitly monitor and support their team members’ work—a widely used mechanism for coordinating tasks in dynamic teamwork settings.^47^ These unintended consequences could be partially alleviated by making the smart glasses easy to use and distraction-free. Nevertheless, how and to what extent utilizing smart glasses impacts teamwork and communication should be further evaluated and quantified.

### Limitations and future research

We did not fully implement and deploy the smart glass technology. Thus, some issues in using this technology in real practice might not be captured. The primary goal of this study was to involve users at the start of the system design process to understand system requirements and design considerations before spending numerous efforts on system implementation. We believe this study approach could lead to better system design and more efficient HIT implementation. In our future work, we will evaluate users’ opinions and perceptions with high-fidelity system prototypes. Another limitation is that due to COVID-19 restrictions, we had to conduct the design workshops online, which could limit participants’ direct interactions with the smart glass device. We followed the best practices shared by other researchers regarding how to achieve the best outcome of conducting web-assisted user studies during the pandemic.^48^ Lastly, we solely relied on 1 methodology (participatory design workshop) to elicit user perceptions. Also, we did not perform member checking on findings. Having study participants review and confirm the results and triangulating the results with more data sources (eg, usability testing) could help validate the study findings.

## CONCLUSION

This study is the first to examine the adoption of smart glasses in EMS through a sociotechnical lens. Involving eventual users (eg, EMS providers) in the early phase of system design and employing a sociotechnical framework allowed us to gain an empirical, in-depth understanding of technical, social, organizational, and human factors that impact the implementation and uptake of this novel technology in EMS. Some user concerns must be addressed by smart glass manufacturers, such as the requirement for durable and weather-proof devices and long-lasting batteries, while other issues related to human-computer interface, workflow, and HIPAA compliances require close collaboration among different stakeholders, including system designers, researchers, policymakers, and medical practitioners. Grounded in our findings, we discussed key design considerations for implementing smart glasses to support EMS work. We believe this study lays out the foundation for future work in designing easy-to-use smart glass applications that can improve EMS workflow and patient care in the field.

## Data Availability

The data underlying this article is shared via datadryad: doi: 10.5061/dryad.3r2280gkw.
