# Correction to: Designing and implementing smart glass technology for emergency medical services: a sociotechnical perspective

**DOI:** 10.1093/jamiaopen/ooad008

**Published:** 2023-02-01

This is a correction to: Zhan Zhang, Noubra Ashika Ramiya Ramesh Babu, Kathleen Adelgais, Mustafa Ozkaynak, Designing and implementing smart glass technology for emergency medical services: a sociotechnical perspective, *JAMIA Open*, Volume 5, Issue 4, December 2022, ooac113, https://doi.org/10.1093/jamiaopen/ooac113

In the originally published version of this manuscript, the name of author Noubra Ashika Ramiya Ramesh Babu contained a typographical error.

This error has been corrected.

